# Activation of ALDH2 with Low Concentration of Ethanol Attenuates Myocardial Ischemia/Reperfusion Injury in Diabetes Rat Model

**DOI:** 10.1155/2016/6190504

**Published:** 2016-10-18

**Authors:** Pin-Fang Kang, Wen-Juan Wu, Yang Tang, Ling Xuan, Su-Dong Guan, Bi Tang, Heng Zhang, Qin Gao, Hong-Ju Wang

**Affiliations:** ^1^Department of Cardiovascular Disease, The First Affiliated Hospital of Bengbu Medical College, Bengbu 233004, China; ^2^Department of Biochemistry and Molecular Biology, Bengbu Medical College, Bengbu 233030, China; ^3^Department of Physiology, Bengbu Medical College, Bengbu 233030, China

## Abstract

The aim of this paper is to observe the change of mitochondrial aldehyde dehydrogenase 2 (ALDH2) when diabetes mellitus (DM) rat heart was subjected to ischemia/reperfusion (I/R) intervention and analyze its underlying mechanisms. DM rat hearts were subjected to 30 min regional ischemia and 120 min reperfusion in vitro and pretreated with ALDH2 activator ethanol (EtOH); cardiomyocyte in high glucose (HG) condition was pretreated with ALDH2 activator Alda-1. In control I/R group, myocardial tissue structure collapse appeared. Compared with control I/R group, left ventricular parameters, SOD activity, the level of Bcl-2/Bax mRNA, ALDH2 mRNA, and protein expressions were decreased and LDH and MDA contents were increased, meanwhile the aggravation of myocardial structure injury in DM I/R group. When DM I/R rats were pretreated with EtOH, left ventricular parameters, SOD, Bcl-2/Bax, and ALDH2 expression were increased; LDH, MDA, and myocardial structure injury were attenuated. Compared with DM + EtOH I/R group, cyanamide (ALDH2 nonspecific blocker), atractyloside (mitoPTP opener), and wortmannin (PI3K inhibitor) groups all decreased left ventricular parameters, SOD, Bcl-2/Bax, and ALDH2 and increased LDH, MDA, and myocardial injury. When cardiomyocyte was under HG condition, CCK-8 activity and ALDH2 protein expression were decreased. Alda-1 increased CCK-8 and ALDH2. Our findings suggested enhanced ALDH2 expression in diabetic I/R rats played the cardioprotective role, maybe through activating PI3K and inhibiting mitoPTP opening.

## 1. Introduction

Diabetes mellitus (DM) is a common metabolic disorder that can affect patient life and survival quality with acute and chronic complications [1–3]. It is associated with a high cardiovascular mortality, and it is also the most common cause of end-stage heart disease [4]. The morbidity and mortality of diabetes show an increasing trend, and the incidence of heart failure after myocardial infarction in diabetic patients is more than 2-3 times than nondiabetic. Myocardial ischemia/reperfusion (I/R) injury can cause cardiac glucose metabolism disorders, calcium overload, and cardiac fibrosis, resulting in accumulation of harmful products, easily leading to apoptosis and necrosis [5]. Therefore, how to protect ischemic myocardium effectively, promote myocardial functional recovery, and reduce myocardial apoptosis become a research hotspot.

Mitochondrial aldehyde dehydrogenase 2 (ALDH2) is one of the key enzymes of alcohol metabolism, which catalyzes the conversion of aldehyde to acetic acid [6]. ALDH2 plays a crucial metabolic role in the detoxification and oxidation of aldehyde, such as inhibiting the production of 4-hydroxynon-2-enal [7–9]. ALDH2 was found to attenuate ethanol exposure-induced myocardial dysfunction, and activation of ALDH2 led to cardiac protection against ischemia and reperfusion injury [10–15]. Previous studies also indicated that, with the development of diabetes, cardiac ALDH2 activity was further decreased, and inhibition of ALDH2 by oxidative stress leads to cardiac dysfunction in diabetes mellitus [16]. What changes of ALDH2 expression that appeared and what mechanisms involved in diabetic rat myocardial I/R injury spark our interest.

At the beginning of I/R, the signal transduction pathways were activated by various means of myocardial protection proactively, especially phosphatidylinositol-3-kinase serine/threonine kinase (PI3K-Akt). PI3K-Akt signaling pathway is an important pathway which regulates cell proliferation, cell division, apoptosis, and other activities. Heart was protected by ischemic preconditioning via activated PI3K-Akt signaling pathway; however, the effect of myocardial protection was abolished by PI3K inhibitor, wortmannin. It is suggested that PI3K-Akt signal transduction pathway was involved in myocardial protection.

Mitochondrial permeability transition pore (mitoPTP) is located in the inner and outer mitochondrial membrane multiprotein complexes, which plays an important role in maintaining mitochondrial membrane potential and protecting the structure and function of mitochondrion [17, 18]. Under physiological condition, mitoPTP is closed, and it has almost no permeability for all mitochondrial metabolites and ions. Opening of mitoPTP is involved in cell death induced by a variety of causes, for example, I/R, endotoxin, and anticancer agents [19]. Opening mitoPTP leads to mitochondrial swelling and outer membrane rupture; the proapoptotic factors and cytochrome C were released into the cytoplasm, inducing energy metabolism unbalance, intracellular calcium overload, and exacerbated ischemic cell damage. The opening of mitoPTP in reperfusion is a sign of myocardial injury from reversible to irreversible [20, 21]. mitoPTP is considered to be the terminal effector of cell death in I/R injury, while delaying or blocking the opening of mitoPTP may reduce I/R injury [22, 23]. A large number of reports suggested that ischemic preconditioning, ischemic postconditioning, and drug pretreatment could protect heart against injury by inhibiting of the opening of mitoPTP [24–26].

Based on above background, we were prompted to investigate the effects of pretreatment with ethanol (EtOH) as a tool to induce ALDH2 activity in myocardial I/R injury of diabetic rats and give drug intervention, including ALDH2 inhibitor cyanimide, PI3K inhibitor wortmannin, and the opener of mitoPTP atractyloside, to determine its underlying mechanisms.

## 2. Materials and Methods

### 2.1. Animals

Male Sprague-Dawley rats (200~250 g) were purchased from the Animal Center of Bengbu Medical College, Anhui. The rats were fed normal chow and had free access to water. Housing was at a constant temperature of (21 ± 1)°C with a fixed 12 h light/dark cycle. All animal procedures were in accordance with United States National Institutes of Health Guide and were approved by the Animal Use and Care Committee of Bengbu Medical College.

### 2.2. Chemicals and Reagents

Streptozotocin (STZ), cyanamide (CYA), wortmannin (Wor), atractyloside (Atr), and Alda-1 were purchased from Sigma (St. Louis, MO, USA). Ethanol (EtOH) was obtained from Bengbu New Chemical Reagent Factory, China. 10% fetal bovine serum was obtained from Zhe Jiang Tianhang Biological corporation, China. Lactate dehydrogenase (LDH), Malondialdehyde (MDA), and superoxide dismutase (SOD) assay kits were purchased from Nanjing Jiancheng Bioengineering Institute, China. CCK-8 assay kit was from Shanghai Bestbio Life Technology, China. The primers used were as follows: for ALDH2 forward: 5′-GTG TTC GGA GAC GTC AAA GA-3′ and reverse 5′-GCA GAG CTT GGG ACA GGT AA-3′, the amplified fragment length was 187 bp; for Bcl-2 forward: 5′-CTG GTG GAC AAC ATC GCT CTG-3′ and reverse: 5′-GGT CTG CTG ACC TCA CTT GTG-3′, the amplified fragment length was 227 bp; for Bax forward: 5′-GGA TCG AGC AGA GAG GAT GG-3′ and reverse: 5′-GCT CAT TGC CGA TAG TGA TGA CT-3′, the amplified fragment length was 464 bp; for *β*-actin forward: 5′-GAT GGT GGG TAT GGG TCA GAA-3′ and reverse: 5′-GGC CAT CTC TTG CTC GAA GTC-3′, the amplified fragment length was 630 bp. Mouse anti-ALDH2, anti-Bcl-2, anti-Bax, and anti-*β*-actin monoclonal antibodies were purchased from Santa Cruz Biotechnology (CA). Goat anti-mouse secondary antibodies were from Boston Co., Ltd., Wuhan, China.

### 2.3. Induction of Diabetes and Experimental Protocol

Diabetes was induced in overnight fasted rats by administering a single intraperitoneal (i.p.) injection of 55 mg/kg streptozotocin (STZ) freshly dissolved in 0.1 mol/L sodium citrate buffer (pH 4.5). The rats in control group were injected with a similar volume of sodium citrate buffer alone. The rats whose fasting blood glucose level was more than 16.7 mmol/L after 72 h of injection were as diabetic [27]. All rats were fed for eight weeks. Animals were randomly divided into control, diabetes (DM), and DM + EtOH groups, respectively. In DM + EtOH group, DM rats were fed with 2.5% EtOH in their drinking water for one week to initiate drinking and then changed to 5% EtOH continuous access through seven weeks.

### 2.4. Ischemia and Reperfusion and Drugs Intervention in Isolated Perfused Heart

**Figure 1:**
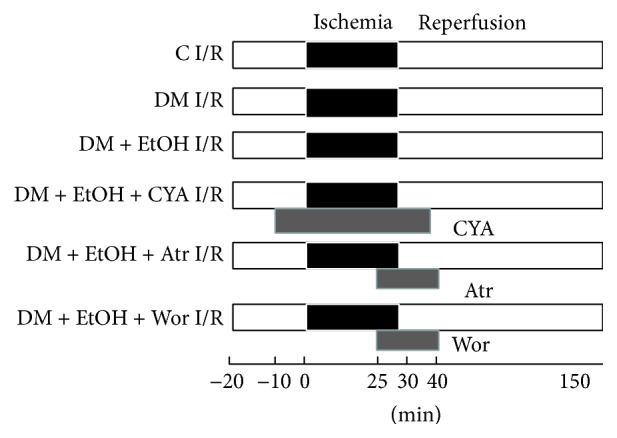
Protocol of various drugs intervention on rat myocardial ischemia/reperfusion model in vitro. C I/R: control ischemia and reperfusion; DM I/R: diabetes rats subjected to myocardial ischemia and reperfusion; DM + EtOH I/R: diabetes + ethanol subjected to myocardial ischemia and reperfusion; DM + EtOH + CYA I/R: diabetes + ethanol + cyanamide subjected to myocardial ischemia and reperfusion; DM + EtOH + Atr I/R: diabetes + ethanol + atractyloside subjected to myocardial ischemia and reperfusion; DM + EtOH + Wor I/R: diabetes + ethanol + wortmannin subjected to myocardial ischemia and reperfusion.

All rat hearts were subjected to regional ischemia and reperfusion intervention (I/R) in vitro. After the rats were anesthetized (chloral hydrate, 4 g/kg, i.p.), hearts were excised rapidly, placed in ice-cold Krebs-Henseleit (K-H) buffer, mounted on a Langendorff apparatus, and perfused at 37°C with K-H buffer. The buffer was equilibrated with 95% O_2_/5% CO_2_ (pH 7.4) and had the following composition (mmol/L): NaCl 118.0, KCl 4.7, CaCl_2_ 1.25, KH_2_PO_4_ 1.2, NaHCO_3_ 25.0, and glucose 11.0. A latex, fluid-filled balloon was introduced into the left ventricle through the left atrial appendage and the balloon catheter was linked to a pressure transducer connected to a data acquisition system (Medlab, Nanjing, China) to assess contractile function. The left ventricular end diastolic pressure (LVEDP) was adjusted to 4~8 mmHg for 30 min. Myocardial infarction (MI) was created by ligation of the left anterior descending artery (LAD). MI was induced by LAD ligation 2~3 mm from the origin with 5-0 silk suture. Then 30 min of regional myocardial ischemia (i.e., MI) followed by 120 min of reperfusion was done in all rats. The cardiac parameters including HR, left ventricular developed pressure (LVDP: difference between left ventricular end systolic pressure and end diastolic pressure), maximal rise/fall rate of left ventricular pressure (±*dp*/*dt*_max_), left ventricular end diastolic pressure (LVEDP), and rate-pressure product (RPP: LVDP × HR) were monitored continuously. The rats were divided into control I/R (*n* = 6), DM I/R (*n* = 6), and DM + EtOH I/R (*n* = 6). Other EtOH treated rats were subjected to three different drugs' intervention, respectively (*n* = 6/group). The ALDH2 inhibitor cyanamide (CYA) at 1 mmol/L was given from 10 min before ischemia to the first 10 min of reperfusion, with total perfusing for 50 min (DM + EtOH + CYA I/R); the mitoPTP opener atractyloside (Atr) at 20 mmol/L was given from the last 5 min of ischemia to the early 10 min of reperfusion, with total perfusing for 15 min (DM + EtOH + Atr I/R). The inhibitor of PI3K, 100 nmol/L wortmannin (Wor), was given at the end ischemia for 5 min and early reperfusion for 10 min (DM + EtOH + Wor I/R) (Figure 1).

### 2.5. Detection of LDH Release in Coronary Flow

Coronary flow was collected at 5 min and 10 min of reperfusion, and LDH release was measured by commercially available kits according to the manufacturer's instructions.

### 2.6. Detection of MDA Content and SOD Activity in Heart Tissue

At the end of the experimental period, 0.1 g heart tissue was homogenized in ice-cold PBS buffer. Malondialdehyde (MDA) content and superoxide dismutase (SOD) activity were measured by commercially available kits according to the manufacturer's instructions.

### 2.7. Detection of Cardiac ALDH2, Bax, and Bcl-2 at mRNA Level by RT-PCR

RT-PCR was used to detect the levels of ALDH2, Bax, and Bcl-2 mRNA expression in heart. Briefly, total RNA was extracted with TRIzol according to the manufacturer's instructions. Two micrograms of total RNA was reverse-transcribed to cDNA, and PCR was performed by a routine method. PCR products were analyzed on 1% agarose gel. Densitometry results for ALDH2, Bax, and Bcl-2 gene were compared with corresponding *β*-actin levels to account for loading differences.

### 2.8. Ultrastructure Observation of Myocardial Cell by Transmission Electron Microscope

Cardiac tissue was dissected and small pieces were fixed with 2.5% glutaraldehyde in 0.1% mol/L cacodylate buffer for 1 h. Ultrathin sections were cut and contrasted with uranylacetate buffer by lead citrate and observed with JEM-1230 transmission electron microscope (JEOL, Japan).

### 2.9. Cell Culture and Drug Treatment

Rat neonatal cardiomyocytes were isolated from 1~2 d old Sprague-Dawley rats by digestion with trypsin. Briefly, ventricular tissue was aseptically removed from neonatal rats, minced, and then digested with mixed protease (0.25% trypsin, 0.2% collagenase II, 100 *μ*g/mL DNA enzyme I, and D-Hanks solution) (Solarbio, Beijing, China) at 37°C in a shaking water bath. The cells were released after the first digestion was discarded, whereas the cells from subsequent digestion were added to an equal volume of Dulbecco's modified Eagle's medium (DMEM) supplemented with 10% fetal bovine serum until all cells were collected. Cells were pelleted by centrifugation at 1000 rpm for 6 min and resuspended in DMEM supplemented 10% fetal bovine serum. The isolated cells were first plated in culture disks of 95% air and 5% CO_2_ at 37°C for 2 h to exclude nonmuscle cells. The suspended cells were then collected and plated at a density of 5 × 10^5^ cells/cm^2^ and maintained under the same conditions as above. The cells were used for outlined experiments after 4~5 days later.

### 2.10. Immunofluorescence Analysis

Cardiomyocytes cultured on coverslips were seeded in 6-well plates and fixed in 4% paraformaldehyde in PBS for 15 min at room temperature, followed by washing with PBS, three times per 5 min. Then, cardiomyocytes were incubated with 0.1% TritonX-100 in PBS for 30 min, followed by washing with PBS, three times per 5 min again. After blocking with 1% bovine serum albumin in PBS for 1 h, the cells were incubated in BSA blocking buffer containing primary antibody-*α*-SMA (1 : 200, Boster, China), and the cells were washed and then incubated in secondary anti-mouse antibody (1 : 200; Boster, China) for 1 h. Furthermore, cardiomyocytes were incubated for 15 min with 4,6-diamidino-2-phenylindole (DAPI; ZSGB, Beijing, China) for nuclear staining, and fluorescence micrographs were obtained using Olympus FSX100 microscope.

### 2.11. Grouping

The cardiomyocytes were divided into 3 groups as follows: control (C) group, HG group, and Alda-1 + HG group. In C group, cardiomyocytes were incubated in DMEM medium including 5.5 mmol/L glucose for 48 h. In HG group, cardiomyocytes were incubated in high glucose medium (25 mmol/L glucose) for 48 h; and in Alda-1 + HG group cardiomyocytes were pretreated with HG and ALDH2 activator Alda-1 (20 *μ*mol/L) for 48 h.

### 2.12. Cell Counting Kit- (CCK-) 8 Assay

Cardiomyocytes (100 *μ*L/well) were seeded into 96-well plate at a density of (0.5~1.0) × 10^4^ cells/mL and incubated at 37°C, 5% CO_2_ overnight. A CCK-8 assay kit was used to detect cell viabilities according to the manufacturer's instructions. Following treatment, test samples and CCK-8 were added to each well 10 *μ*L, respectively. After 1~4 h incubation, cells viability was determined by measuring the absorbance at 450 nm using microplate reader. The activity levels were determined according to the manufacturer's instructions.

### 2.13. Detection of Cardiac and Cardiomyocyte ALDH2 Protein Expression by Western Blot

The ALDH2 protein in cardiac tissue and cardiomyocyte was detected by western blot [1]. Anti-ALDH2 (1 : 500) antibody was used. Mouse anti-*β*-actin antibody (1 : 500) was used as an internal control. The immunoblots were exposed to X-ray film and analyzed with a digital image system.

### 2.14. Statistical Analysis

All values are expressed as mean ± SD. Statistical comparisons were performed by one-way analysis of variance and the Newman-Keuls test. Differences of *p* < 0.05 were regarded as significant.

## 3. Results

### 3.1. Changes of Ventricular Hemodynamic Parameters

In C I/R group, compared with baseline, LVDP, ±*dp*/*dt*_max_, and RPP were decreased, and LVEDP was increased during ischemia period; during reperfusion period, LVDP, ±*dp*/*dt*_max_, and RPP were lower and LVEDP was higher. During ischemia and reperfusion period, in contrast to C I/R rat, in DM I/R rat, LVDP, ±*dp*/*dt*_max_, and RPP were decreased, and LVEDP was increased; compared with DM I/R group, LVDP, ±*dp*/*dt*_max_, and RPP were increased, and LVEDP was decreased in DM + EtOH I/R group; compared with DM + EtOH I/R group, in DM + EtOH + CYA I/R group, LVDP, ±*dp*/*dt*_max_, and RPP were furtherly decreased, and LVEDP was increased; the changes in DM + EtOH + Atr I/R and DM + EtOH + Wor I/R groups were similar to DM + EtOH + CYA I/R group (Table 1). There were no differences among the three groups, in DM + EtOH + cyanamide + I/R versus DM + EtOH + wortmannin + I/R versus DM + EtOH + atractyloside + I/R.

### 3.2. Changes of LDH Content

The changes of LDH content in different groups were shown in Table 2. In contrast to C I/R group, in DM I/R group rats, LDH release was increased. Compared with DM I/R group, LDH release was decreased in DM + EtOH I/R group. Compared with DM + EtOH I/R group, LDH release was increased in DM + EtOH + CYA I/R, DM + EtOH + Atr I/R, and DM + EtOH + Wor I/R groups (Table 2).

### 3.3. Changes of MDA Content and SOD Activity in Heart Tissue

**Figure 2:**
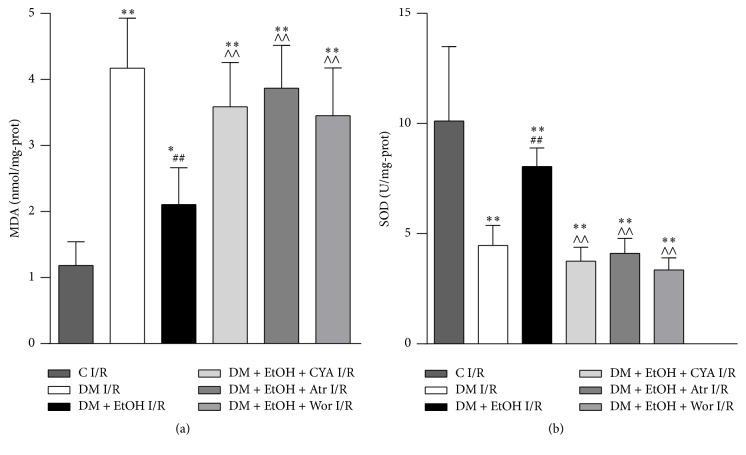
The levels of MDA (a) and SOD (b) of rats myocardium in each group (mean ± SD *n* = 5). ^*∗*^ *p* < 0.05, ^*∗∗*^ *p* < 0.01 versus C I/R; ^##^ *p* < 0.01 versus DM I/R; ^∧∧^ *p* < 0.01 versus DM + EtOH I/R.

Compared with C I/R group, in DM I/R group, MDA content was increased, and SOD activity was decreased. Compared with DM I/R group, in DM + EtOH I/R group, MDA content was decreased, accompanied with the increase of SOD activity. Compared with DM + EtOH I/R group, MDA content was increased, and SOD activity was decreased in DM + EtOH + CYA I/R, DM + EtOH + Atr I/R, and DM + EtOH + Wor I/R groups (Figure 2).

### 3.4. Change of Bax and Bcl-2 at mRNA Level in Heart

**Figure 3:**
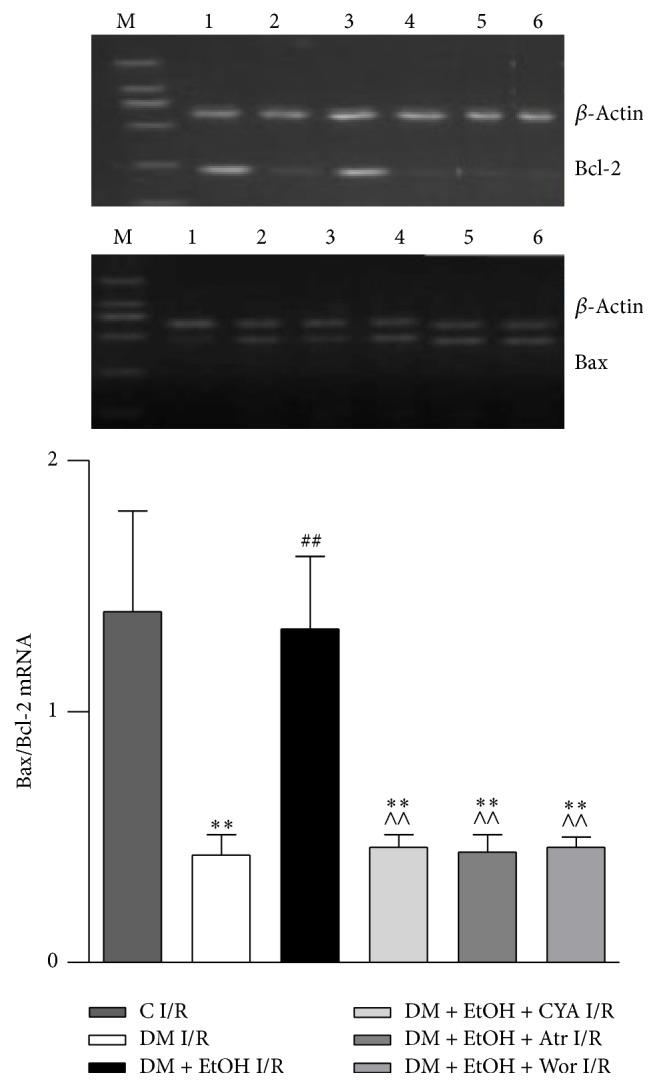
The expressions of Bcl-2 and Bax mRNA of heart tissue in rats (mean ± SD of 5 separate experiments). ^*∗∗*^ *p* < 0.01 versus C I/R; ^##^ *p* < 0.01 versus DM I/R; ^∧∧^ *p* < 0.01 versus DM + EtOH I/R. (1) C I/R; (2) DM I/R; (3) DM + EtOH I/R; (4) DM + EtOH + CYA I/R; (5) DM + EtOH + Atr I/R; (6) DM + EtOH + Wor I/R.

Compared with C I/R group, Bax mRNA was increased; Bcl-2 mRNA and the ratio of Bcl-2/Bax were significantly decreased in DM I/R group. Compared with DM I/R group, in DM + EtOH I/R group, Bax mRNA was decreased; Bcl-2 mRNA and the ratio of Bcl-2/Bax were increased. Compared with DM + EtOH I/R group, Bax mRNA was decreased; Bcl-2 mRNA and the ratio of Bcl-2/Bax were decreased in DM + EtOH + CYA I/R, DM + EtOH + Atr I/R, and DM + EtOH + Wor I/R groups (Figure 3).

### 3.5. Change of ALDH2 mRNA and Protein Level in Heart

**Figure 4:**
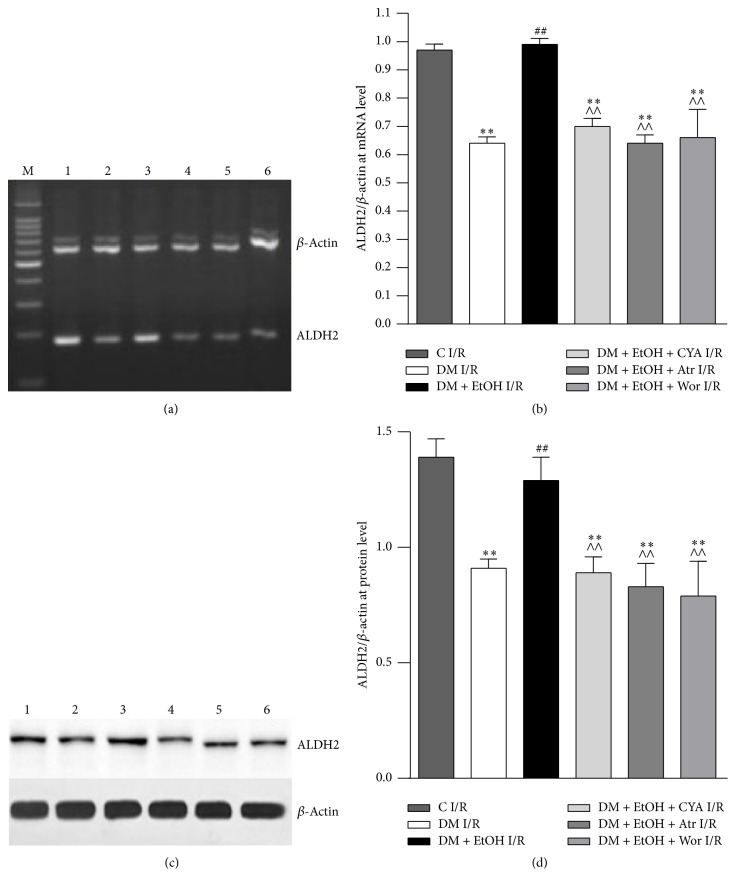
Expressions of ALDH2 at mRNA (a, b) and protein (c, d) level of heart tissue in rats (mean ± SD of 5 separate experiments). ^*∗∗*^ *p* < 0.01 versus C I/R; ^##^ *p* < 0.01 versus DM I/R; ^∧∧^ *p* < 0.01 versus DM + EtOH I/R. (1) C I/R; (2) DM I/R; (3) DM + EtOH I/R; (4) DM + EtOH + CYA I/R; (5) DM + EtOH + Atr I/R; (6) DM + EtOH + Wor I/R.

Compared with C I/R group, ALDH2 mRNA and protein expressions were significantly decreased in DM I/R group. Compared with DM I/R group, ALDH2 mRNA and protein expressions were increased significantly in DM + EtOH I/R group; compared with DM + EtOH I/R group, ALDH2 mRNA and protein expressions were decreased in DM + EtOH + CYA I/R and DM + EtOH + Atr I/R and DM + EtOH + Wor I/R groups (Figures 4(a), 4(b), 4(c), and 4(d)).

### 3.6. CCK-8 Assay and ALDH2 Protein Level in Cardiomyocyte

**Figure 5:**
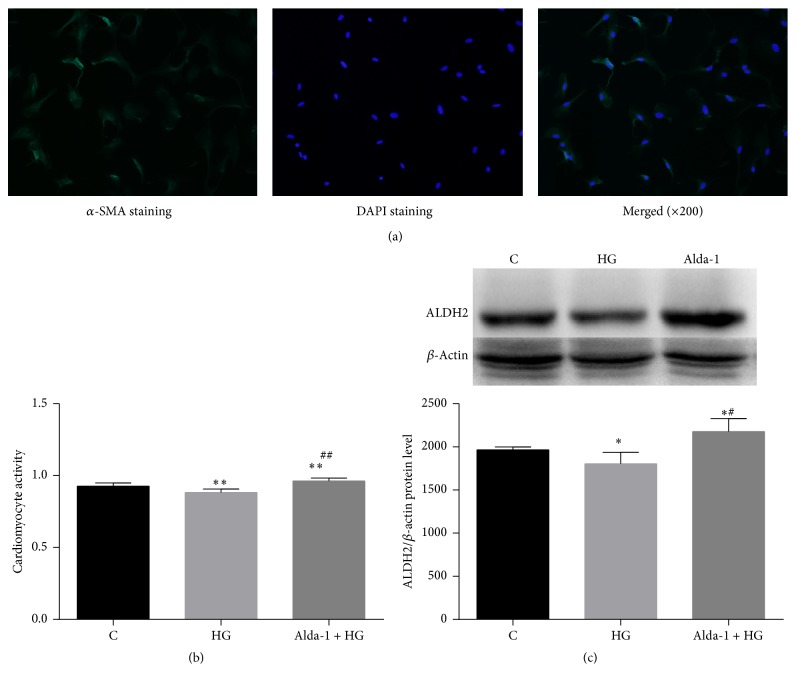
Identify cardiomyocytes by immunofluorescence (a), CCK-8 activity (b), and expressions of ALDH2 protein level of cardiomyocyte in rats (c) (mean ± SD of five separate experiments). ^*∗*^ *p* < 0.05, ^*∗∗*^ *p* < 0.01 versus C, ^#^ *p* < 0.05, and ^##^ *p* < 0.01 versus HG.

Compared with C group, cardiomyocyte activity and ALDH2 protein expressions were significantly decreased in HG group. Compared with HG group, cardiomyocyte activity and ALDH2 protein expression were increased significantly in Alda-1 + HG group (Figures 5(a), 5(b), and 5(c)).

### 3.7. Ultrastructural Changes of Myocardial Cell

**Figure 6:**
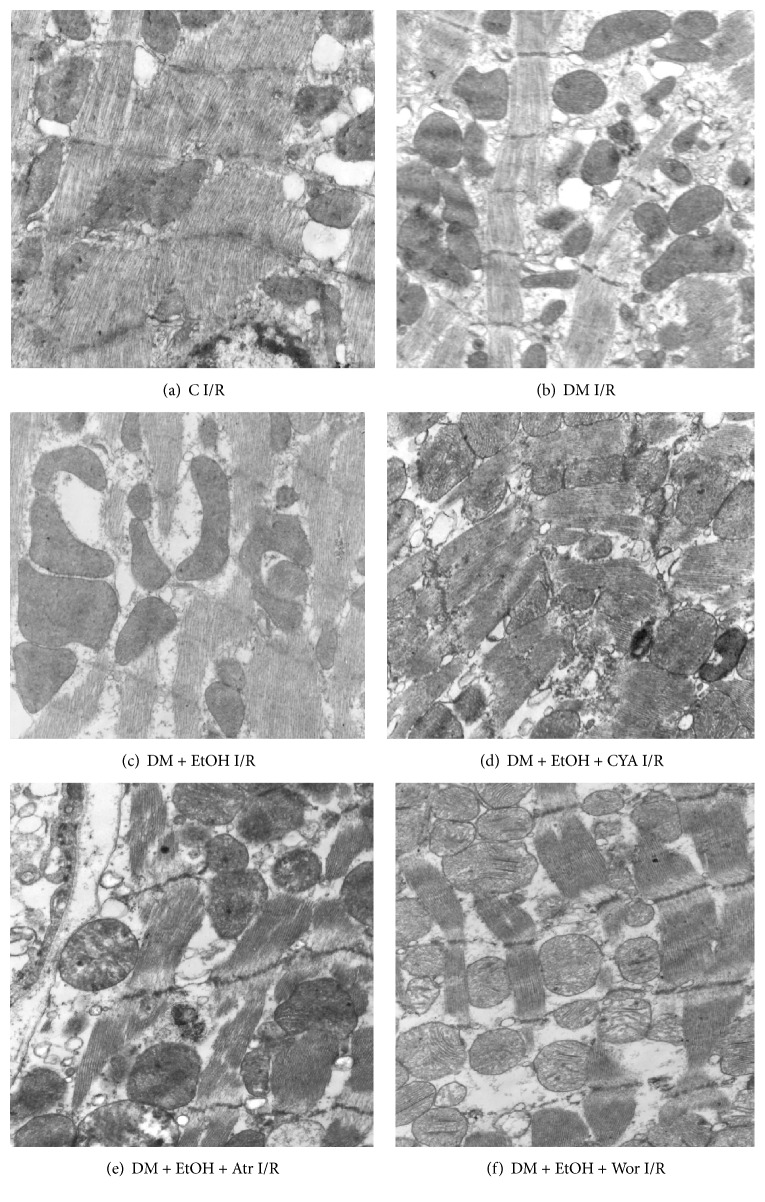
Changes of rat myocardium ultrastructural organization in each group (magnification: 10K).

In C I/R group, mitochondrial dense membrane was uncompleted, mild swelling, and myocardial myofilament was fractured, no large tracts of dissolution; in DM I/R group, mitochondrial swelling was aggravated, myocardial cytoplasmic muscle fiber was ruptured, and large areas of dissolution and vacuolation occurred. Compared with DM I/R group, in DM + EtOH I/R group, the degree of derangement of the cardiac myofilament and mitochondrial swelling was lessened; compared with DM + EtOH I/R group, in DM + EtOH + CYA I/R, DM + EtOH + Atr I/R, and DM + EtOH + Wor I/R groups, the injury was increased (Figure 6).

## 4. Discussion

In the current study, we mimicked myocardial ischemia/reperfusion (I/R) injury in diabetic rats. Compared with C I/R group, LDH levels were increased, cardiac systolic and diastolic dysfunction were aggravated, as indicated by the decreases of LVDP, ±*dp*/*dt*_max_, and RPP and increases of LVEDP, cardiac oxidative stress injury was aggravated, as indicated by the decrease of SOD activity and increase of MDA content, and the expression of Bcl-2 mRNA was decreased while Bax mRNA was increased, also indicating that myocyte apoptosis was aggravated. Meanwhile, we also found that myocardial ultrastructure was damaged in DM I/R group. Myocardial ALDH2 at mRNA and protein levels were decreased in DM I/R group. When diabetes rats were pretreated with low concentration of EtOH, which was used as a tool to induce ALDH2 activity, LDH level was decreased; whereas LVDP, ±*dp*/*dt*_max_, RP, SOD activity and Bcl-2 mRNA level were increased, MDA content and Bax mRNA level were decreased. Also, the injury to myocardial ultrastructure was attenuated. The effect of EtOH was associated with increased myocardial ALDH2 mRNA and protein levels. When the diabetic rats were treated with CYA, Wor, or Atr after EtOH intervention, the injury was increased, and ALDH2 activity was reduced, and LDH level was decreased; whereas LVDP, ±*dp*/*dt*_max_, RPP, SOD activity and Bcl-2 mRNA level were increased, MDA content and Bax mRNA levels were decreased. At the same time, the injury to myocardial ultrastructure was aggravated in DM + EtOH + CYA I/R group, and the changes in DM + EtOH + Atr I/R group and DM + EtOH + Wor I/R group were similar to DM + EtOH + CYA I/R group. These results suggested that decreases in ALDH2 expression may play a key role in myocardial I/R injury in diabetic rats. Upregulation of ALDH2 can protect the heart against myocardial I/R injury, and the mechanism may be through activation of PI3K-Akt signaling pathway and inhibiting mitoPTP opening.

Cardiovascular complications remain the leading cause of diabetes related mortality and morbidity. The oxidative stress of diabetes can increase myocardial I/R injury; myocardial I/R injury produced excessive amounts of reactive oxygen species and increasingly aggravated the injury of myocardial reperfusion. Several reports in recent years had discussed the association between ALDH2 and myocardial injury. Overexpression of ALDH2 offers myocardial protection maybe against alcohol-induced cardiac tissue and cellular injury. Enhanced ALDH2 activity by the direct effect of ALDH2 activator-1 or ethanol preconditioning led to cardiac protection against I/R injury; on the other hand, myocardial I/R injury may be exacerbated after ALDH2 knockout in mice [28, 29]. Earlier findings from our group also indicated that activation of ALDH2 with ethanol attenuated diabetes-induced myocardial injury in rats [27]. However, there are few researches on the relationship of myocardial ALDH2 and diabetes myocardial ischemia/reperfusion injury. Recent evidence revealed that ALDH2 prevented ROS-induced vascular contraction in angiotensin-II induced hypertensive mice [30]. ALDH2 overexpression attenuated hyperoxia-induced cell death in lung epithelial cells through reduction of ROS, activation of ERK/MAPK, and PI3K-Akt signaling pathways [31]. Thus far, there have been no reports about the changes of ALDH2 in diabetes myocardial I/R injury, and the underlying cellular mechanisms are not clear.

Therefore, in the present study, we firstly sought to determine whether ALDH2 expression also changed in diabetes myocardial I/R injury model. In previous study, we observed that, with the progression of diabetes, myocardial ALDH2 expression was further decreased accompanying decreased ventricular function, and activation of ALDH2 can decrease diabetes- induced myocardial injury. In the present study, we used diabetic I/R model and observed cardiac systolic and diastolic function was destroyed, and myocardial ultrastructure was damaged accompanied with the aggravation of oxidative stress. At the same time, myocardial ALDH2 at mRNA and protein levels were decreased, and LDH release in the coronary effluent was increased. It is suggested that the expression of ALDH2 was related to the cardiac I/R injury induced by diabetes.

ALDH2 as an antioxidant enzyme may be easily inactivated by free radicals. Recently, ALDH2 was identified as a target for oxidative modification during glyceryl trinitrate tolerance [32, 33]; and ALDH2 activity was correlated inversely with cardiac infarct size in rat hearts subjected to ischemia and reperfusion ex vivo [34]. We used low concentration of EtOH as a tool to induce ALDH2 activity to investigate whether upregulation of ALDH2 expression could benefit recovery from myocardial function; it is likely that increasing ALDH2 expression can attenuate the happening of oxidative stress and the destroying of myocardial function. These findings suggest that, compared with diabetic I/R group, ALDH2 overexpression can exert the protective effect against cardiac I/R injury of diabetes. However, the ALDH2 inhibitor CYA, which was involved in ALDH2, conferred protection against cardiac I/R injury, and LDH release was increased, and the expressions of ALDH2 at mRNA and protein level were decreased. The result showed that ALDH2 overexpression reconciled diabetes-induced contractile dysfunction, whereas cardioprotection elicited by ALDH2 was blocked by the inhibitor CYA. Further study is required to better elucidate the mechanism of ALDH2. Mitochondrial permeability transition pore (mitoPTP) is a nonselective highly conductive channel which exists between mitochondrial inner and outer membranes, playing an important role in apoptosis. The opening of mitoPTP is an ultimate target of all kinds of damage; ischemia/reperfusion induced mitoPTP to open and also induced the overload of intracellular calcium, accumulation of ROS, and increase in PH and other factors [35]. Argaud et al. reported in the rabbit myocardial ischemia model that when the rabbits were given preconditioning, postconditioning, and mitoPTP inhibitor, respectively, during reperfusion, the effects were similar in reducing myocardial infarct size, and the tolerance of myocardial mitochondria was decreased for calcium overload in I/R injury. However, the myocardial mitochondria resistance was increased after postconditioning or mitoPTP inhibitor treatment [36]. These studies indicated that postconditioning may be via inhibiting the opening of mitoPTP resulting in reducing the myocardial damage by overload of calcium. It is suggested that the myocardial protection of preconditioning and postconditioning was involved in suppression of open mitoPTP. In the present paper, we further investigate the role of ALDH2 in the diabetic rat hearts. In our study, we used the specific openers of mitoPTP atractyloside (Atr). The result suggested that Atr canceled the protection of EtOH; indeed, we found the ALDH2 mRNA and protein levels were reduced; meanwhile, cardiac LV contractile function was declining and myocardial ultrastructure was damaged accompanied with the aggravation of oxidative stress. Zhou et al. reported on hydrogen peroxide-induced myocardial injury model. Low concentration of ethanol can inhibit the opening of mitoPTP induced by oxidative stress via GSK-3*β* pathway and PI3K/Akt; however, high concentration of ethanol-fed rats can induce the cell apoptosis and opening of mitoPTP [37, 38]. These results displayed that promoting the opening of mitoPTP might be via inhibiting ALDH2 generation against its protection.

It is well known that PI3K/Akt signaling is a key mediator of cell survival. Akt is also implicated in the cardioprotection for cardiac myocyte survival [39]. However, the role of Akt signaling in regulation of diabetic rat myocardial I/R injury is not clear. Activation of Akt was found to rescue the cardiac mechanical function defect induced by ER stress [40]. In the study, we used the PI3K/Akt inhibitor wortmannin (Wor); compared with DM + EtOH I/R group, ALDH2 mRNA and protein levels were decreased; meanwhile, cardiac LV contractile function and myocardial ultrastructure were damaged accompanied with the aggravation of oxidative stress. Data depicted that ALDH2 induced cell survival was blocked by inhibition of PI3K. These results suggested that the protection of ALDH2 was at least partially mediated by PI3K/Akt pathway. However, further study is required to better elucidate the mechanism of ALDH2 cardiac protection.

I/R injury is a complex process and results in oxidative stress in myocardium, such as O_2_^−^ and H_2_O_2_ accumulating and resulting in cellular toxicity, finally due to imbalance between production and removal of ROS. The survival myocardial cells after myocardial infarction were more prone to calcium overload and therefore more susceptible to I/R injury; accumulating evidence proved that overproduction of ROS triggered myocyte apoptosis by upregulating proapoptotic members of Bcl-2 family. In addition, investigators have shown that Bcl-2 gene family is known to modulate the permeability of the mitochondrial membrane and the release of cytochrome C [41]. Therefore, we investigated whether the expressions of Bax and Bcl-2 genes are related to ALDH2 induced protection in diabetic myocardial I/R injury. In our study, we detected Bax and Bcl-2 at mRNA levels. The result showed that the expression of cardiac Bcl-2 at mRNA level was decreased, the expression of Bax at mRNA level was increased, Bcl-2/Bax mRNA was decreased in DM I/R group, and LDH release in the coronary effluent of diabetic rats was increased. Meanwhile, ALDH2 mRNA and protein were decreased. However, compared with DM I/R group, the ratio of Bax to Bcl-2 mRNA and LDH levels were decreased in DM + EtOH I/R group, and the expressions of ALDH2 mRNA and protein were increased, which suggested that the increase of myocardial cells apoptosis may be associated with the decrease of ALDH2. To further prove our hypothesis, via given drug intervention, ALDH2 inhibitor CYA, PI3K inhibitor Wor, and the opener of mitoPTP Atr, the result suggests the ratio of Bax to Bcl-2 mRNA was decreased; meanwhile, ALDH2 levels were decreased accompanied with the changes of cardiac LV contractile function, oxidative stress, and myocardial ultrastructure.

To verify whether cardiac ALDH2 directly participates in diabetes-induced cardiomyocyte injury, in this study, we also applied high glucose induced cardiomyocyte injury to mimic diabetes. We found that, in cardiomyocytes model, compared with C group, CCK-8 activity and the ALDH2 protein expression were decreased in HG group; when cardiomyocytes were pretreated with Alda-1, the specific activator of ALDH2 in HG condition, CCK-8 activity was increased accompanied with the increase of ALDH2 protein expression. It is suggested that direct activation of cardiomyocyte ALDH2 attenuated high glucose induced myocardial injury. It is worthwhile to note that there were some limitations in our study. In cardiomyocyte model, we only observed the protection of enhanced ALDH2 expression in HG condition. To better understand the mechanism involved, we will investigate the effect of cardiomyocytes ALDH2 in hypoxia and reoxygenation status and further validate our hypothesis.

In conclusion, activation of ALDH2 participates in the regulation of diabetic I/R injury induced cardiac myocyte apoptosis via mitochondrial pathway. ALDH2 may play an antiapoptotic effect through decreasing the ratio of Bax/Bcl-2, activation of PI3K/Akt signaling pathway, and inhibiting mitoPTP opening. Promoting ALDH2 expression may become a new pathway for the clinical treatment DM with CHD patients, and further studies are necessary to confirm this hypothesis.

## Figures and Tables

**Table 1:** Hemodynamic parameters in the isolated perfused rat hearts subjected to ischemia and reperfusion (I/R) (mean ± SD, *n* = 5).

Variable	Baseline	Ischemia	Reperfusion		
		30 min	5 min	10 min	120 min
LVDP (% of baseline )					
C I/R	100 ± 0	59 ± 13	63 ± 13	65 ± 14	58 ± 4
DM I/R	100 ± 0	33 ± 7^*∗∗*^	42 ± 12^*∗*^	48 ± 5^*∗∗*^	32 ± 5^*∗∗*^
DM + EtOH I/R	100 ± 0	59 ± 15^##^	93 ± 12^*∗∗*##^	90 ± 7^*∗∗*##^	58 ± 13^##^
DM + EtOH + CYA I/R	100 ± 0	36 ± 7^*∗∗*∧∧^	44 ± 8^*∗∗*∧∧^	44 ± 8^*∗∗*∧∧^	35 ± 9^*∗∗*∧∧^
DM + EtOH + Atr I/R	100 ± 0	38 ± 8^*∗∗*∧^	48 ± 8^*∗*∧∧^	47 ± 8^*∗∗*∧∧^	30 ± 4^*∗∗*∧∧^
DM + EtOH + Wor I/R	100 ± 0	39 ± 6^*∗∗*∧∧^	47 ± 8^*∗*∧∧^	44 ± 7^*∗∗*∧∧^	32 ± 3^*∗∗*∧∧^
LVEDP (% of baseline)					
C I/R	100 ± 0	101 ± 1	116 ± 2	120 ± 10	177 ± 11
DM I/R	100 ± 0	151 ± 15^*∗∗*^	177 ± 14^*∗∗*^	159 ± 9^*∗∗*^	262 ± 27^*∗∗*^
DM + EtOH I/R	100 ± 0	104 ± 7^##^	143 ± 6^*∗∗*##^	141 ± 8^*∗∗*##^	182 ± 14^##^
DM + EtOH + CYA I/R	100 ± 0	155 ± 11^*∗∗*∧∧^	182 ± 11^*∗∗*∧∧^	160 ± 12^*∗∗*∧∧^	254 ± 20^*∗∗*∧∧^
DM + EtOH + Atr I/R	100 ± 0	147 ± 20^*∗∗*∧∧^	166 ± 3^*∗∗*∧∧^	166 ± 5^*∗∗*∧∧^	251 ± 16^*∗∗*∧∧^
DM + EtOH + Wor I/R	100 ± 0	168 ± 19^*∗∗*∧∧^	176 ± 7^*∗∗*∧∧^	170 ± 11^*∗∗*∧∧^	281 ± 45^*∗∗*∧∧^
+*dp*/*dt*_max_ (% of baseline)					
C I/R	100 ± 0	64 ± 10	80 ± 15	78 ± 12	55 ± 8
DM I/R	100 ± 0	48 ± 8	43 ± 9^*∗∗*^	46 ± 5^*∗∗*^	34 ± 6^*∗∗*^
DM + EtOH I/R	100 ± 0	60 ± 14	91 ± 17^##^	91 ± 19^##^	62 ± 11^##^
DM + EtOH + CYA I/R	100 ± 0	47 ± 7	46 ± 6^*∗∗*∧∧^	47 ± 6^*∗∗*∧∧^	32 ± 7^*∗∗*∧∧^
DM + EtOH + Atr I/R	100 ± 0	48 ± 6	45 ± 2^*∗∗*∧∧^	47 ± 4^*∗∗*∧∧^	34 ± 7^*∗∗*∧∧^
DM + EtOH + Wor I/R	100 ± 0	49 ± 9	43 ± 6^*∗∗*∧∧^	44 ± 4^*∗∗*∧∧^	33 ± 5^*∗∗*∧∧^
−*dp*/*dt*_max_ (% of baseline)					
C I/R	100 ± 0	59 ± 13	59 ± 10	59 ± 1	41 ± 5
DM I/R	100 ± 0	42 ± 9^*∗∗*^	42 ± 6^*∗∗*^	46 ± 9^*∗∗*^	31 ± 4^*∗∗*^
DM + EtOH I/R	100 ± 0	62 ± 5^##^	85 ± 10^*∗∗*##^	84 ± 12^*∗∗*##^	48 ± 6^*∗*##^
DM + EtOH + CYA I/R	100 ± 0	45 ± 4^*∗*∧∧^	46 ± 7^*∗*∧∧^	44 ± 4^*∗∗*∧∧^	35 ± 4^*∗*∧∧^
DM + EtOH + Atr I/R	100 ± 0	43 ± 5^*∗∗*∧∧^	49 ± 5^*∗*∧∧^	45 ± 4^*∗∗*∧^	33 ± 4^*∗∗*∧∧^
DM + EtOH + Wor I/R	100 ± 0	46 ± 4^*∗*∧^	44 ± 5^*∗∗*∧∧^	43 ± 4^*∗∗*∧∧^	30 ± 5^*∗∗*∧∧^
RPP (% of baseline)					
C I/R	100 ± 0	59 ± 6	62 ± 17	62 ± 8	33 ± 3
DM I/R	100 ± 0	26 ± 7^*∗∗*^	32 ± 8^*∗∗*^	28 ± 7^*∗∗*^	18 ± 4^*∗∗*^
DM + EtOH I/R	100 ± 0	55 ± 13^##^	76 ± 7^*∗*##^	79 ± 19^*∗*##^	37 ± 6^##^
DM + EtOH + CYA I/R	100 ± 0	29 ± 5^*∗∗*∧∧^	28 ± 5^*∗∗*∧∧^	25 ± 6^*∗∗*∧∧^	15 ± 5^*∗∗*∧∧^
DM + EtOH + Atr I/R	100 ± 0	28 ± 4^*∗∗*∧∧^	34 ± 6^*∗∗*∧∧^	31 ± 6^*∗∗*∧∧^	16 ± 4^*∗∗*∧∧^
DM + EtOH + Wor I/R	100 ± 0	32 ± 8^*∗∗*∧∧^	30 ± 3^*∗∗*∧∧^	25 ± 4^*∗∗*∧∧^	18 ± 2^*∗∗*∧∧^

^*∗*^ *p* < 0.05, ^*∗∗*^ *p* < 0.01 versus C I/R; ^##^ *p* < 0.01 versus DM I/R; ^∧^ *p* < 0.05, ^∧∧^ *p* < 0.01 versus DM + EtOH I/R.

**Table 2:** Effect of different drugs on LDH release in the coronary effluent in the isolated rat hearts subjected to ischemia and reperfusion (I/R) (mean ± SD, *n* = 5).

Group	5 min (U/L)	10 min (U/L)
C I/R	66.08 ± 7.60	60.50 ± 6.93
DM I/R	154.58 ± 16.50^*∗∗*^	129.58 ± 22.23^*∗∗*^
DM + EtOH I/R	77.92 ± 11.62^##^	61.58 ± 5.63^##^
DM + EtOH + CYA I/R	139.83 ± 20.41^*∗∗*∧∧^	107.92 ± 27.42^*∗∗*∧∧^
DM + EtOH + Atr I/R	137.17 ± 22.14^*∗∗*∧∧^	112.67 ± 24.02^*∗∗*∧∧^
DM + EtOH + Wor I/R	136.25 ± 29.42^*∗∗*∧∧^	113.75 ± 18.40^*∗∗*∧∧^

^*∗∗*^ *p* < 0.01 versus C I/R; ^##^ *p* < 0.01 versus DM I/R; ^∧∧^ *p* < 0.01 versus DM + EtOH I/R.
